# Unraveling the relationship between gut microbiota and site-specific endometriosis: a Mendelian randomization analysis

**DOI:** 10.3389/fmicb.2024.1363080

**Published:** 2024-07-04

**Authors:** Yuanyuan Tang, Jiangbing Yang, Fu Hang, Hui Huang, Li Jiang

**Affiliations:** ^1^Department of Reproductive Center, Maternal and Child Health Hospital of Guangxi Zhuang Autonomous Region, Nanning, China; ^2^School of Basic Medical Sciences, Guangxi Medical University, Nanning, China; ^3^Department of Reproductive Center, First Affiliated Hospital of Guangxi Medical University, Nanning, China; ^4^Guangxi Henbio Biotechnology Co., Ltd., Nanning, China

**Keywords:** Mendelian randomization, endometriosis, gut microbiota, infertility, single-nucleotide polymorphisms, pathogenesis

## Abstract

**Background:**

Although numerous studies have illustrated the connection between gut microbiota and endometriosis, a conspicuous gap exists in research focusing on the pathogenesis of endometriosis at various sites and its linkage with infertility.

**Methods:**

In this study, we used a two-sample Mendelian randomization analysis to investigate the effect of gut microbiota on the development of endometriosis in different regions, including the uterus, ovary, fallopian tube, pelvic peritoneum, vagina, and rectovaginal septum, as well as the intestine. Additionally, we explored the correlation between gut microbiota and endometriosis-induced infertility. Genetic associations with gut microbes were obtained from genome-wide association study (GWAS) datasets provided by the MiBioGen consortium, whereas endometriosis-related GWAS data were sourced from the FinnGen dataset. In our analysis, single-nucleotide polymorphisms were used as instrumental variables, with the primary estimation of the causal effect performed via the inverse variance weighting method. Our sensitivity analyses incorporated heterogeneity tests, pleiotropy tests, and the leave-one-out method.

**Results:**

We identified associations at the genus level between four bacterial communities and endometriosis. Subsequently, several associations between the gut microbiota and various subtypes of endometriosis at different anatomical sites were recognized. Specifically, three genera were linked with ovarian endometriosis, six genera were associated with tubal endometriosis, four genera showed links with pelvic peritoneum endometriosis, five genera were connected with vaginal and rectovaginal septum endometriosis, and seven genera demonstrated linkages with intestinal endometriosis. Additionally, one genus was associated with adenomyosis, and three genera exhibited associations with endometriosis-induced infertility.

**Conclusion:**

Our study elucidates associations between gut microbiota and site-specific endometriosis, thereby augmenting our understanding of the pathophysiology of endometriosis. Moreover, our findings pave the way for potential therapeutic strategies targeting gut microbiota for individuals grappling with endometriosis-related infertility.

## 1 Introduction

Endometriosis, an estrogen-dependent chronic inflammatory disease (Ozkan et al., 2008), is characterized by functional endometrial glands and stroma outside the uterine cavity (Vercellini et al., 2014). It commonly affects adjacent tissues and organs, such as the ovaries, uterosacral ligaments, uterine wall muscles, and pelvic peritoneum. However, its presence in extrapelvic organs, such as the gastrointestinal tract, urinary tract, lungs, nasal mucosa, and brain, is rare (Machairiotis et al., 2013). According to the existing literature, endometriosis affects approximately 2–10% of women and has a prevalence of as high as 50% in women experiencing infertility (Becker et al., 2022). Its main clinical manifestations are chronic pelvic pain, dysmenorrhea, dyspareunia, dyschezia, and complications including infertility. These symptoms can affect patients’ fertility, mental health, and social functioning. Given that endometriosis has an insidious onset, atypical clinical symptoms, and lacks specific diagnostic markers, its definitive diagnosis may be delayed for 4–11 years (Taylor et al., 2021). The high recurrence rates after surgery and the side effects of medication further complicate the treatment of this disease. Therefore, additional research is urgently needed to explore the pathogenesis of endometriosis to provide a theoretical basis and guidance for clinical treatment strategies.

Various hypotheses regarding the origin of ectopic endometrial-like tissue exist. They include coelomic metaplasia, induction, retrograde menstruation, and lymphatic spread theories (Talwar et al., 2022). Among these hypotheses, Sampson’s theory of retrograde menstruation is widely accepted (Sampson, 1927, 1940). This theory proposes that viable fragments of endometrial tissue reflux through the fallopian tubes to the ovaries and peritoneal cavity during menstruation, leading to the development of ectopic endometrial lesions (Bulun et al., 2019). However, even though over 90% of women of reproductive age experience retrograde menstruation, only approximately 10% develop endometriosis. This situation suggests that Sampson’s theory alone cannot fully explain the pathogenesis of endometriosis. Therefore, composite theories combining implantation and lymphatic spread have been proposed (Javert, 1949). Considering the varied locations of endometriotic tissue, endometriosis exhibits heterogeneity. All the above hypotheses are based on the understanding that different genetic, hormonal, inflammatory, immune, and environmental factors substantially contribute to endometriosis (Wang et al., 2020; Saunders and Horne, 2021). However, these factors alone cannot explain the pathogenesis of endometriosis at different ectopic sites (Lagana et al., 2017). The pathogenesis of this disease, such as its origin, remains poorly understood.

The human gut microbiome, often referred to as the “second human genome,” encompasses approximately 10^13^ to 10^14^ microorganisms, primarily consisting of Bacteroidetes, Firmicutes, Actinobacteria, and Proteobacteria. Changes in the composition and function of the gut microbiota can influence intestinal permeability, digestion, metabolism, and immune responses due to their role in immune regulation and metabolic processes. Recent studies have demonstrated a close association between gut microbiota and the onset and development of various diseases, including diabetes (Yang et al., 2021), irritable bowel syndrome (IBS) (Salmeri et al., 2023b), colon cancer (Novello et al., 2019), and polycystic ovary syndrome (Liang et al., 2021). Up to 90% of patients with endometriosis report gastrointestinal symptoms, such as bloating, nausea, constipation, diarrhea, and vomiting (Xholli et al., 2023), in addition to gynecological symptoms. This situation suggests a potential link between gut health and endometriosis. In recent years, the relationship between gut microbiota and endometriosis has attracted increasing attention from researchers (Chadchan et al., 2021; Talwar et al., 2022). The possible mechanisms through which gut microbiota imbalance influences the pathogenesis of endometriosis include the following: (1) Involvement of the gut microbiota in immune-mediated chronic inflammation regulation. Gut microbiota imbalance disrupts immune responses, leading to inflammation and the impaired immune clearance of endometrial fragments, thereby promoting the development of endometriotic lesions (Tang et al., 2024). (2) Bacterial contamination hypothesis (Khan et al., 2018): this hypothesis involves the lipopolysaccharide (LPS)/Toll-like receptor (TLR) 4 cascade. The gut microbiota is believed to influence the generation of serum LPS, triggering an inflammatory cascade reaction, stimulating the release of immune cells and inflammatory factors in the body, establishing a chronic inflammatory state, and accelerating the development of endometriosis (Anderson, 2019). (3) Involvement of the gut microbiota in estrogen metabolism changes: The collection of genes encoding estrogen-metabolizing enzymes in the gut microbiota is known as the “estrobolome” (Ervin et al., 2019). The gut microbiota secretes β-glucuronidase, which can deconjugate estrogen and increase the reabsorption of free estrogen, hence raising serum estrogen levels and promoting the growth of ectopic endometrial lesions (Kwa et al., 2016; Baker et al., 2017). Moreover, gut microbiota interacts with hormones, such as estrogen, androgen, and insulin, thus playing a crucial role in the female reproductive endocrine system (Qi et al., 2021). (4) The gut-brain axis of endometriosis. The gut microbiota regulates bidirectional communication with the central nervous system through the gut-brain axis (Salmeri et al., 2023b). The gut microbiota promotes central sensitization to chronic pain by regulating neuroinflammatory responses. This effect may also be the basis of chronic pain in endometriosis. Given that gut microbiota imbalance has numerous adverse effects on the body, correcting this imbalance to restore normal function may aid in the future treatment of endometriosis. However, the efficacy of gut microbiota preparations in treating endometriosis remains controversial. In observational studies, the relationship between gut microbiota and endometriosis can easily be confounded by factors, such as age, environment, and lifestyle (Rinninella et al., 2019). Therefore, further exploration and refinement are needed to identify characteristic microbial changes in the gut of patients with endometriosis and their causal relationships.

Mendelian randomization (MR) has recently gained considerable clinical relevance in assessing causal relationships between exposure factors and diseases (Grover et al., 2017). It serves as an effective methodology for determining causal associations between exposure and outcome by utilizing genetic variants that are strongly associated with exposure as instrumental variables (IVs). MR can be likened to a randomized controlled trial because it has a low susceptibility to confounding factors, providing a high level of evidence. Previous studies have applied MR to investigate the link between gut microbiota and endometriosis, identifying *Anaerotruncus*, *Olsenella*, and *Oscillospira* as potential risk factors for endometriosis (Liang et al., 2023). Despite the current understanding, no studies have yet elucidated the mechanisms underlying the association between gut microbiota and site-specific endometriosis. Consequently, this study was designed to investigate comprehensively the potential causal relationship between gut microbiota and endometriosis in different regions, including the uterus, ovary, fallopian tube, pelvic peritoneum, vagina, and rectovaginal septum, as well as the intestine. In addition, this research aims to explore the correlation between gut microbiota and endometriosis-related infertility using a two-sample MR analysis. The findings of this investigation are expected to enhance our understanding of the underlying mechanisms of endometriosis and provide innovative insights into the development of therapeutic strategies for treating infertility associated with endometriosis.

## 2 Materials and methods

### 2.1 Exposure to gut microbiota

We utilized single-nucleotide polymorphisms (SNPs) associated with human gut microbiota as IVs. The data from a comprehensive genome-wide association study (GWAS) dataset provided by the MiBioGen consortium were utilized. This GWAS dataset incorporated 16S fecal microbiome data and expansive genotype information from 24 study cohorts worldwide (Kurilshikov et al., 2021). The project included a total of 18,340 participants, comprising 16,632 adults and adolescents, as well as 1708 children, representing diverse ethnic backgrounds, including European, Middle-Eastern, East Asian, American Hispanic/Latin, and African American backgrounds, with Europeans accounting for 72.3% of the cohort. The gut microbiota data consisted of 211 taxa with a relative abundance exceeding 1%, encompassing 9 phyla, 16 classes, 20 orders, 35 families, and 131 genera. After we excluded 15 taxa of unknown groups (12 genera and three families), 119 genera were included in the analysis of this study. The GWAS data relevant to gut microbiota were obtained from http://www.mibiogen.org.

### 2.2 GWAS summary data for endometriosis

We obtained data on endometriosis from the FinnGen cohort, accessed through the OPEN GWAS platform,^1^ with the GWAS ID finn-b-N14-ENDOMETRIOSIS. The diagnosis of endometriosis in patients was based on the International Classification of Diseases, 10th Edition. This dataset included 77,257 European female participants, consisting of 8,288 cases and 68,969 controls. Patients with endometriosis included those with adenomyosis (2,372 cases), ovarian endometriosis (3,231 cases), tubal endometriosis (116 cases), pelvic peritoneal endometriosis (2,953 cases), vaginal and rectovaginal septum endometriosis (1,360 cases), intestinal endometriosis (177 cases), endometriosis in cutaneous scars (unknown cases), and unspecified/other endometriosis (1,435 cases). We compiled GWAS data from the FinnGen cohort to investigate the potential causal relationship between gut microbiota and site-specific endometriosis. These data pertain to endometriosis occurring at diverse locations, including the uterus, ovary, fallopian tube, pelvic peritoneum, vagina, and rectovaginal septum, as well as the intestine. Furthermore, we sought to explore the role of gut microbiota in endometriosis-related infertility. Patients with endometriosis complicated by infertility included 1,593 cases and 70,651 controls. These data can be accessed through the GWAS IDs provided in the R package TwoSampleMR (version 0.5.7). The GWAS IDs are listed in the IEU OpenGWAS database. Refer to Table 1 for detailed information.

**Table 1:** Summary of genome-wide association studies (GWAS) datasets in our study.

Phenotype	Source	Population	Sample Size	No. of cases	No. of controls	GWAS ID
Gut microbiome	MiBioGen	Multi-ancestry	18,340	—	—	—
Endometriosis	FinnGen	European	77,257	8,288	68,969	finn-b-N14_ENDOMETRIOSIS
Ovarian endometriosis	FinnGen	European	72,200	3,231	68,969	finn-b-N14_ENDOMETRIOSIS_OVARY
Tubal endometriosis	FinnGen	European	69,085	116	68,969	finn-b-N14_ENDOMETRIOSIS_FALLOPIAN_TUBE
Pelvic peritoneum endometriosis	FinnGen	European	71,922	2,953	68,969	finn-b-N14_ENDOMETRIOSIS_PELVICPERITONEUM
Vaginal and rectovaginal septum endometriosis	FinnGen	European	70,329	1,360	68,969	finn-b-N14_ENDOMETRIOSIS_RECTPVAGSEPT_VAGINA
Intestinal endometriosis	FinnGen	European	69,146	177	68,969	finn-b-N14_ENDOMETRIOSIS_INTESTINE
Adenomyosis	FinnGen	European	71,341	2,372	68,969	finn-b-N14_ENDOMETRIOSIS_UTERUS
Endometriosis-related infertility	FinnGen	European	72,244	1,593	70,651	finn-b-N14_ENDOMET_INFERT

### 2.3 IV selection

We examined the association between gut microbiota and endometriosis by considering gut microbiota as the exposure and endometriosis as the outcome. We implemented several quality control steps to screen the IVs to ensure the precision and stability of our analysis. First, we selected SNPs that showed a significant association with gut microbes as IVs (*p* < 1 × 10^−5^). In the second step, we applied a clumping window of 10,000 kb and an *r*^2^ threshold of 0.001 to exclude SNPs in linkage disequilibrium. For the third step, we replaced missing SNPs with those with high linkage (*R*^2^ > 0.8) and removed SNPs without sufficient substitution sites. In the fourth step, we set a screening threshold of 0.3 for minor allele frequency. In the fifth step, we excluded palindrome SNPs to avoid any misrepresentation in strand direction or allelic coding. Furthermore, we calculated the F-statistic by using the formula *F* = R^2^ × (*N* − 2)/(1 − *R*^2^) to assess the strength of IVs. A high *F*-statistic value indicates a low bias from weak IVs, and a value exceeding 10 is generally considered acceptable (Burgess and Thompson, 2011).

### 2.4 MR analysis

**Figure 1:**
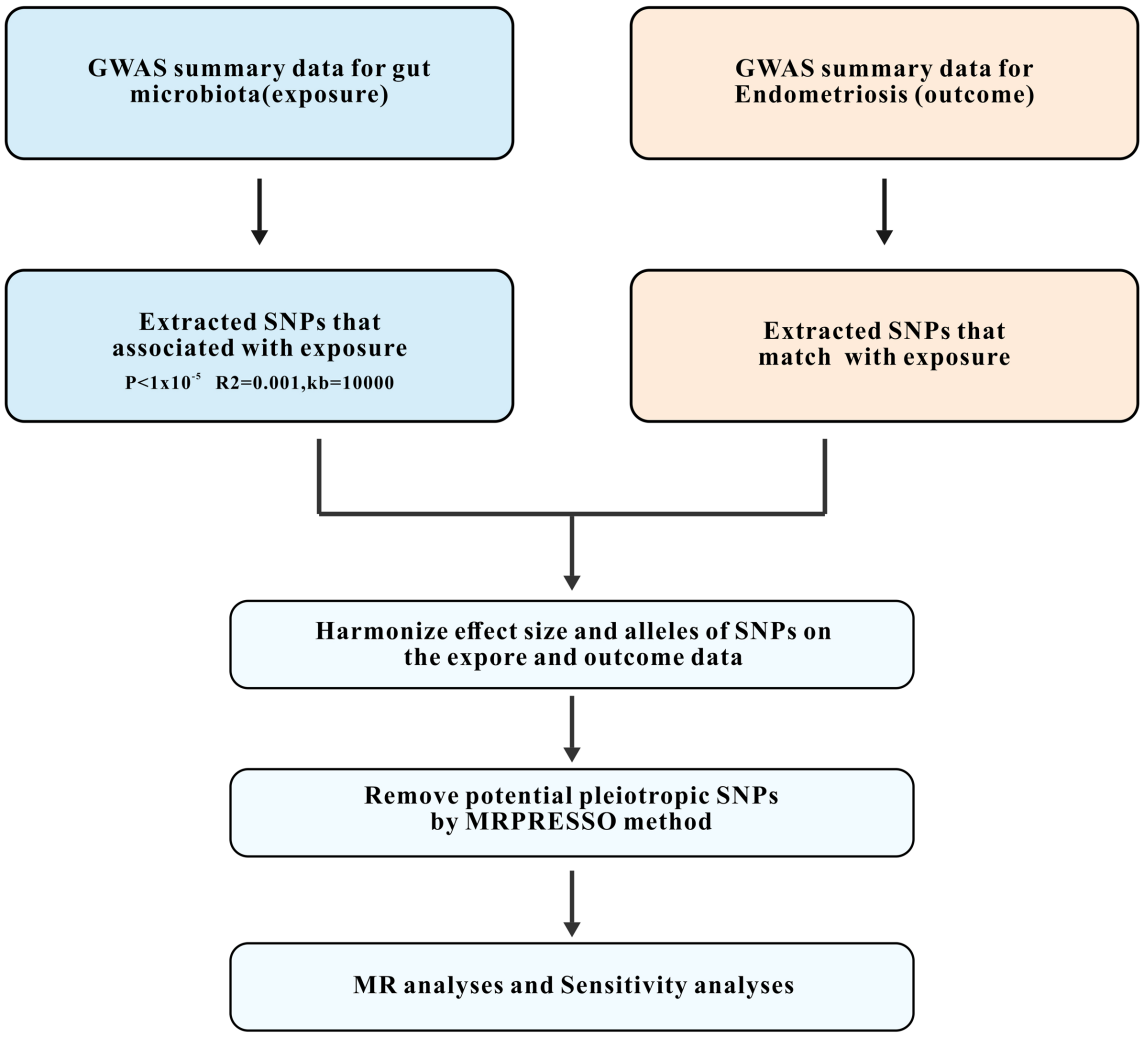
Study design and workflow. GWAS, the genome-wide association study; SNPs, single-nucleotide polymorphisms; MR, Mendelian randomization.

Our MR analysis adhered to three fundamental assumptions. First, the IVs should correlate significantly with the exposure variable (gut microbiota). Second, the effect of IVs on the outcome variable (endometriosis) should not be confounded by other variables. Third, the IVs should influence the outcome solely through the exposure variable, indicating the absence of horizontal pleiotropy. An overview of the study design is illustrated in Figure 1. A total of 119 genera of gut microbiota were selected based on the GWAS data, and IVs were screened for each genus. Two-sample MR analyses were then conducted independently to assess the causal effect of gut microbiota on endometriosis at specific locations and endometriosis combined with infertility. The Wald ratio was used to estimate the association between the identified IVs and the outcome for a single IV (Burgess et al., 2017). The inverse-variance weighted (IVW) method was predominantly used for multiple IVs. IVW combines the Wald ratios of outcomes for each SNP to obtain an estimate while accounting for excessive dispersion (Burgess et al., 2013). Additionally, supplemental verification methods, including weighted modal estimation (Hartwig et al., 2017), simple mode estimation (Hartwig et al., 2017), MR-Egger regression (Bowden et al., 2015), and weighted median estimation (Bowden et al., 2016) were used.

### 2.5 Sensitivity analysis

We conducted a sensitivity analysis to assess the robustness of the findings. This approach involved leave-one-out analysis, wherein we systematically evaluated one SNP at a time to evaluate its influence on results. Heterogeneity was assessed by using Cochran’s *Q* statistic, with a *p*-value below 0.05 indicating the presence of significant heterogeneity (Greco et al., 2015). Furthermore, we used the MR-Egger intercept test to detect potential pleiotropy in the data, with a *p*-value below 0.05, suggesting the presence of pleiotropic effects (Burgess and Thompson, 2017).

### 2.6 Statistical analysis

All procedures linked to the selection and quality control of IVs were conducted using the R packages TwoSampleMR (version 0.5.7) and Mendelian Randomization Pleiotropy RESidual Sum and Outlier (MR-PRESSO) in R software (version 4.2.1). A *p*-value of less than 0.05 was considered to demonstrate a statistically significant correlation.

## 3 Results

### 3.1 SNP selection

Initially, we identified SNPs associated with gut microbiota at the genus level in consideration of significance levels of *p* < 1 × 10^5^. A total of 1,213 SNPs were included as IVs. Among these SNPs, we observed 49 that were significantly associated with endometriosis. In the subsequent phase, we focused on investigating the direct associations between intestinal flora and site-specific endometriosis. Our findings revealed 14, 20, 63, and 32 independent SNPs associated with adenomyosis, ovarian endometriosis, tubal endometriosis, and pelvic peritoneum endometriosis, respectively. Additionally, we identified 46, 69, and 34 independent SNPs associated with rectovaginal septum and vaginal endometriosis, intestinal endometriosis, and endometriosis accompanied by infertility, respectively (Supplementary Table S1). After we implemented rigorous quality control measures, all IVs exhibited *F*-statistics exceeding 10, indicating the absence of weak IVs. The MR-PRESSO global test did not detect any evidence of pleiotropy (*p* > 0.05). Finally, we identified pleiotropic SNPs using the MR-PRESSO outlier test and excluded them from further analysis, resulting in no indications of horizontal pleiotropy in the re-evaluated IVs (*p*-values >0.05 for the MR-PRESSO global test and MR-Egger regression).

### 3.2 Gut microbiota and endometriosis

**Figure 2:**
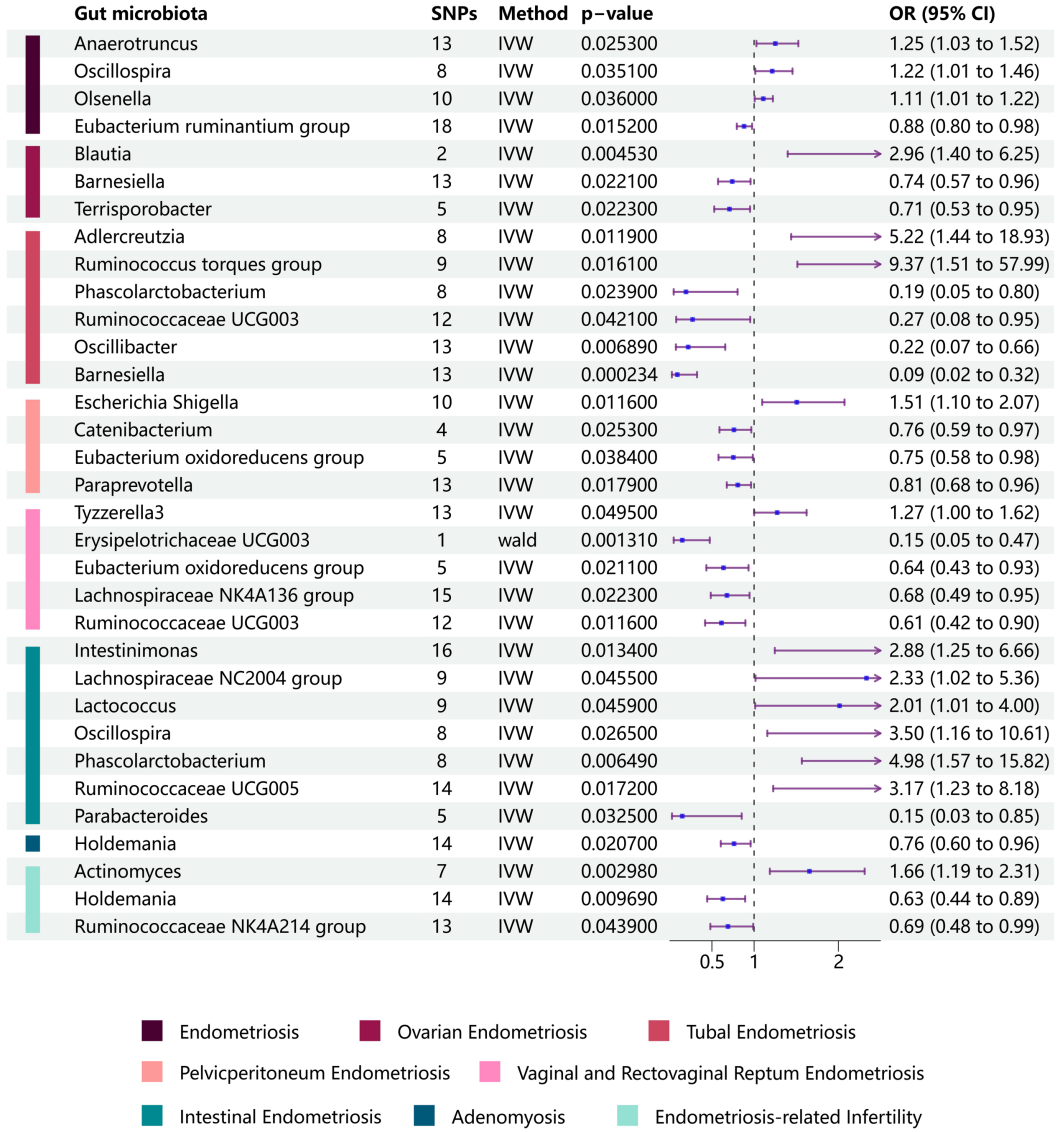
Forest plots of the Mendelian randomization analysis of site-specific endometriosis. Relationships between gut microbiota and site-specific endometriosis were estimated using the IVM method. The purple bars represent the 95% confidence interval of IVM estimates. An OR >1 indicates an increased risk, while an OR <1 indicates a decreased risk (IVM, inverse-variance weighting; OR, odds ratio).

We conducted a two-sample MR analysis to investigate the potential role of gut microbiota in the development of endometriosis. Our findings revealed an association between endometriosis and four bacterial communities at the genus level. Specifically, we identified the *Eubacterium ruminantium* group as a protective factor against endometriosis [odds ratio (OR): 0.881, 95% confidence interval (CI): 0.795–0.976, *p* = 0.0152], whereas we identified the genera *Anaerotruncus* (OR: 1.252, 95% CI: 1.028–1.525, *p* = 0.0253), *Olsenella* (OR: 1.109, 95% CI: 1.007–1.223, *p* = 0.0360), and *Oscillospira* (OR: 1.215, 95% CI: 1.014–456, *p* = 0.0351) to be risk factors (Figure 2 and Table 2).

**Table 2:** MR analysis and Sensitivity analysis of gut microbiome in endometriosis and endometriosis-related infertility (*p* < 1 × 10^−5^).

Outcomes	Bacterial genera (exposure)	nSNPs	Methods	OR (95% CI)	Beta	Se	*p*-value	Heterogeneity		MR-PRESSO	Horizontal pleiotropy		
								Cochran’sQ	*p*-value	*p*-value	Egger intercept	Se	*p*-value
Endometriosis	*Anaerotruncus*	13	MR Egger	0.85 (0.49–1.47)	−0.17	0.28	0.57	11.47	0.41	0.34	0.03	0.02	0.17
			IVW	1.25 (1.03–1.53)	0.23	0.10	0.03	13.76	0.32				
			Weighted median	1.24 (0.97–1.59)	0.22	0.13	0.08						
			Simple mode	1.21 (0.87–1.66)	0.19	0.17	0.28						
			Weighted mode	1.20 (0.87–1.66)	0.18	0.16	0.29						
	*Eubacterium ruminantium* group	18	MR Egger	0.93 (0.62–1.24)	−0.13	0.1	0.47	12.73	0.69	0.83	0.00	0.02	0.98
			IVW	0.88 (0.79–0.98)	−0.13	0.05	0.02	12.73	0.75				
			Weighted median	0.88 (0.80–1.07)	−0.08	0.07	0.31						
			Simple mode	0.96 (0.74–1.25)	−0.04	0.13	0.78						
			Weighted mode	0.96 (0.77–1.19)	−0.04	0.11	0.70						
	*Olsenella*	10	MR Egger	1.03 (0.75–1.40)	0.03	0.16	0.86	7.12	0.52	0.59	0.01	0.02	0.63
			IVW	1.11 (1.01–1.22)	0.10	0.05	0.04	7.37	0.60				
			Weighted median	1.11 (0.97–1.26)	0.10	0.07	0.13						
			Simple mode	1.12 (0.89–1.41)	0.12	0.12	0.35						
			Weighted mode	1.04 (0.86–1.26)	0.04	0.10	0.71						
	*Oscillospira*	8	MR Egger	0.96 (0.45–2.06)	−0.04	0.39	0.92	2.88	0.24	0.73	0.02	0.04	0.55
			IVW	1.21 (1.01–1.46)	0.19	0.09	0.04	3.27	0.86				
			Weighted median	1.17 (0.93–1.47)	0.15	0.12	0.19						
			Simple mode	1.14 (0.78–1.66)	0.13	0.19	0.53						
			Weighted mode	1.12 (0.80–1.58)	0.11	0.17	0.54						
Adenomyosis	*Holdemania*	14	MR Egger	0.85 (0.43–1.67)	−0.16	0.35	0.64	9.72	0.64	0.858	−0.01	0.03	0.75
			IVW	0.76 (0.61–0.96)	−0.27	0.12	0.02	9.83	0.71				
			Weighted median	0.84 (0.60–1.16)	−0.18	0.17	0.28						
			Simple mode	0.83 (0.45–1.54)	−0.18	0.31	0.57						
			Weighted mode	0.87 (0.48–1.55)	−0.14	0.30	0.64						
Endometriosis-related infertility	*Actinomyces*	7	MR Egger	0.88 (0.39–1.99)	−0.12	0.42	0.78	0.97	0.96	0.79	0.08	0.05	0.16
			IVW	1.66 (1.19–2.31)	0.50	0.17	0.00	3.73	0.71				
			Weighted median	1.41 (0.89–2.24)	0.35	0.23	0.14						
			Simple mode	1.40 (0.66–2.99)	0.34	0.38	0.41						
			Weighted mode	1.30 (0.74–2.29)	0.27	0.29	0.39						
	*Holdemania*	14	MR Egger	0.36 (0.13–0.99)	−1.03	0.52	0.07	18.94	0.09	0.09	0.06	0.05	0.27
			IVW	0.63 (0.52–1.11)	−0.46	0.18	0.01	21.06	0.07				
			Weighted median	0.76 (0.44–0.89)	−0.27	0.19	0.16						
			Simple mode	0.75 (0.42–1.33)	−0.29	0.29	0.34						
			Weighted mode	0.75 (0.43–1.31)	−0.28	0.28	0.34						
	*Ruminococcaceae NK4A214* group	13	MR Egger	0.31 (0.09–1.01)	−1.18	0.61	0.08	7.23	0.78	0.71	0.06	0.04	0.19
			IVW	0.69 (0.48–0.99)	−0.37	0.18	0.04	9.19	0.69				
			Weighted median	0.68 (0.41–1.10)	−0.39	0.25	0.12						
			Simple mode	0.71 (0.31–1.64)	−0.34	0.42	0.44						
			Weighted mode	0.68 (0.32–1.45)	−0.39	0.39	0.34						

### 3.3 Gut microbiota and ovarian endometriosis

We performed additional analyses to explore the relationships between gut microbiota and specific subtypes of endometriosis and to understand the mechanisms underlying the development of endometriosis at different locations. For ovarian endometriosis, we identified the *Blautia* genus as detrimental (OR: 2.958, 95% CI: 1.399–6.253, *p* = 0.0045), whereas the *Barnesiella* (OR: 0.741, 95% CI: 0.573–0.958, *p* = 0.0221) and *Terrisporobacter* genera (OR: 0.708, 95% CI: 0.527–0.952, *p* = 0.0223) were found to be protective.

### 3.4 Gut microbiota and fallopian tube endometriosis

*Adlercreutzia* and *Ruminococcus torques* (OR: 9.37, 95% CI: 1.51–57.99, *p* = 0.0161) were associated with an increased risk for fallopian tube endometriosis. Conversely, *Barnesiella* (OR: 0.09, 95% CI: 0.01–0.45, *p* = 0.0002), *Oscillibacter* (OR: 0.22, 95% CI: 0.07–0.66, *p* = 0.0069), *Phascolarctobacterium* (OR: 0.19, 95% CI: 0.05–0.80, *p* = 0.0239), and *Ruminococcaceae UCG003* (OR: 0.27, 95% CI: 0.08–0.95, *p* = 0.0421) were associated with a decreased risk for fallopian tube endometriosis.

### 3.5 Gut microbiota and pelvic peritoneum endometriosis

The *Escherichia-Shigella* genus was associated with an increased risk for pelvic peritoneum endometriosis (OR: 1.51, 95% CI: 1.10–2.07, *p* = 0.0116). By contrast, *Catenibacterium* (OR: 0.76, 95% CI: 0.60–0.97, *p* = 0.0253), *Eubacterium oxidoreducens* group (OR: 0.75, 95% CI: 0.58–0.98, *p* = 0.0384), and *Paraprevotella* (OR: 0.81, 95% CI: 0.68–0.95, *p* = 0.0179) were associated with a decreased risk for pelvic peritoneum endometriosis.

### 3.6 Gut microbiota and vaginal/rectovaginal septum endometriosis

We also identified associations between gut microbiota and vaginal/rectovaginal septum endometriosis. Specifically, *Tyzzerella3* was associated with an increased risk, whereas *Erysipelotrichaceae UCG003*, *Eubacterium oxidoreducens group*, *Lachnospiraceae NK4A136 group*, and *Ruminococcaceae UCG003* were associated with a decreased risk for vaginal/rectovaginal septum endometriosis.

### 3.7 Gut microbiota and intestinal endometriosis

In the case of intestinal endometriosis, *Intestinimonas*, *Lachnospiraceae NC2004 group*, *Lactococcus*, *Oscillospira*, *Phascolarctobacterium*, and *Ruminococcaceae UCG005* were associated with an increased risk, whereas *Parabacteroides* was associated with a decreased risk. These findings provide valuable insights into the potential role of gut microbiota in the development and progression of various forms of endometriosis (Figure 2 and Supplementary Table S2).

### 3.8 Gut microbiota and adenomyosis

We also examined adenomyosis, a specific type of endometriosis characterized by the infiltration of endometrial glands and stroma into the myometrium. In our findings, *Holdemania* genus emerged as a protective factor against adenomyosis (OR: 0.762, 95% CI: 0.605–0.959, *p* = 0.0207).

### 3.9 Association between gut microbiota and endometriosis-related infertility

**Figure 3:**
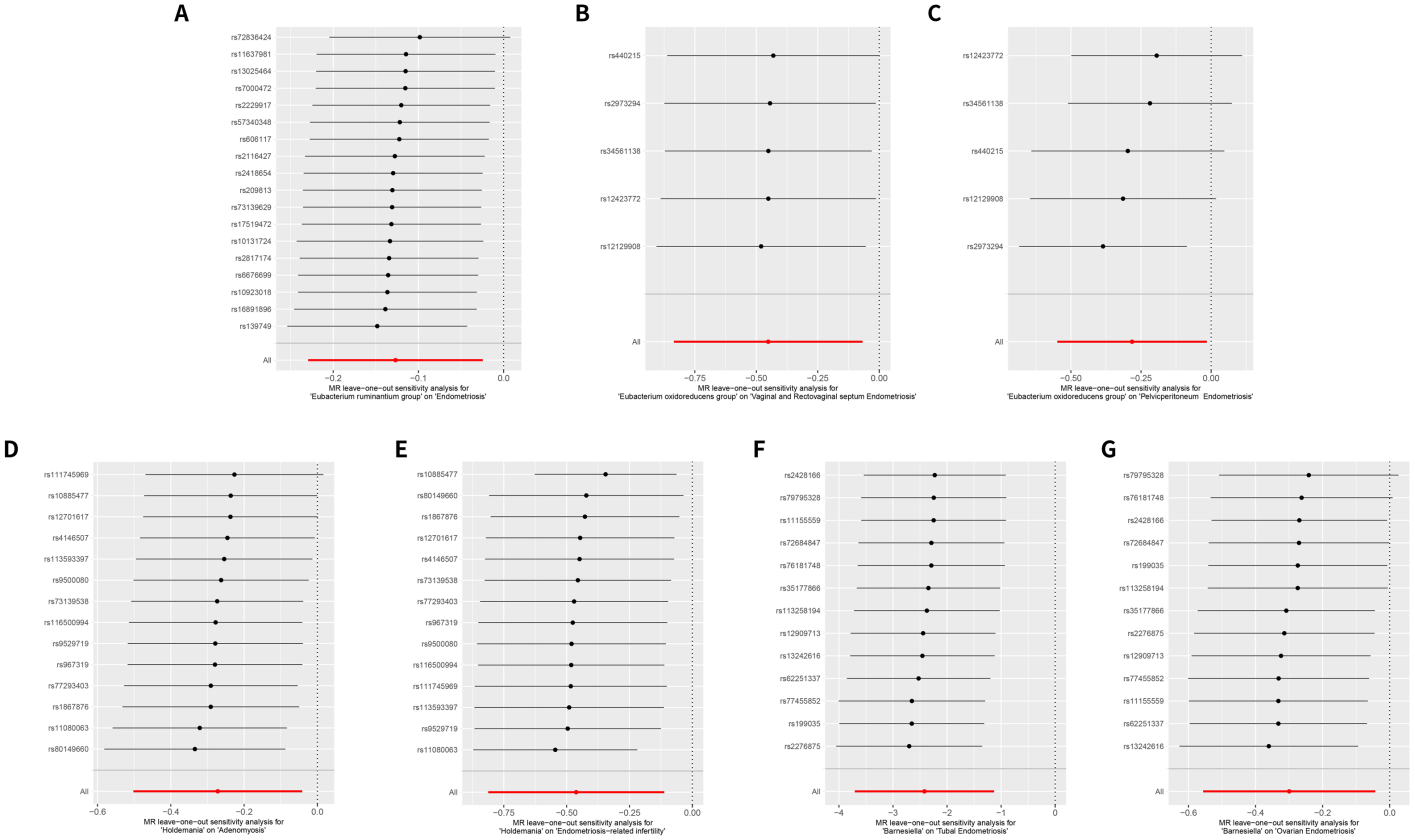
Leave-one-out sensitivity analysis reveals a consistent protective role of the gut microbiome at the same genus in different subtypes of endometriosis. **(A)** MR leave-one-out sensitivity analysis for *Eubacterium ruminantium* group on endometriosis. **(B)** MR leave-one-out sensitivity analysis for *Eubacterium oxidoreducens* group on vaginal and rectovaginal septum endometriosis. **(C)** MR leave-one-out sensitivity analysis for *Eubacterium oxidoreducens* group on pelvic peritoneum endometriosis. **(D)** MR leave-one-out sensitivity analysis for *Holdemania* on adenomyosis. **(E)** MR leave-one-out sensitivity analysis for *Holdemania* on endometriosis-related infertility. **(F)** MR leave-one-out sensitivity analysis for *Barnesiella* on tubal endometriosis. **(G)** MR leave-one-out sensitivity analysis for *Barnesiella* on ovarian endometriosis.

**Figure 4:**
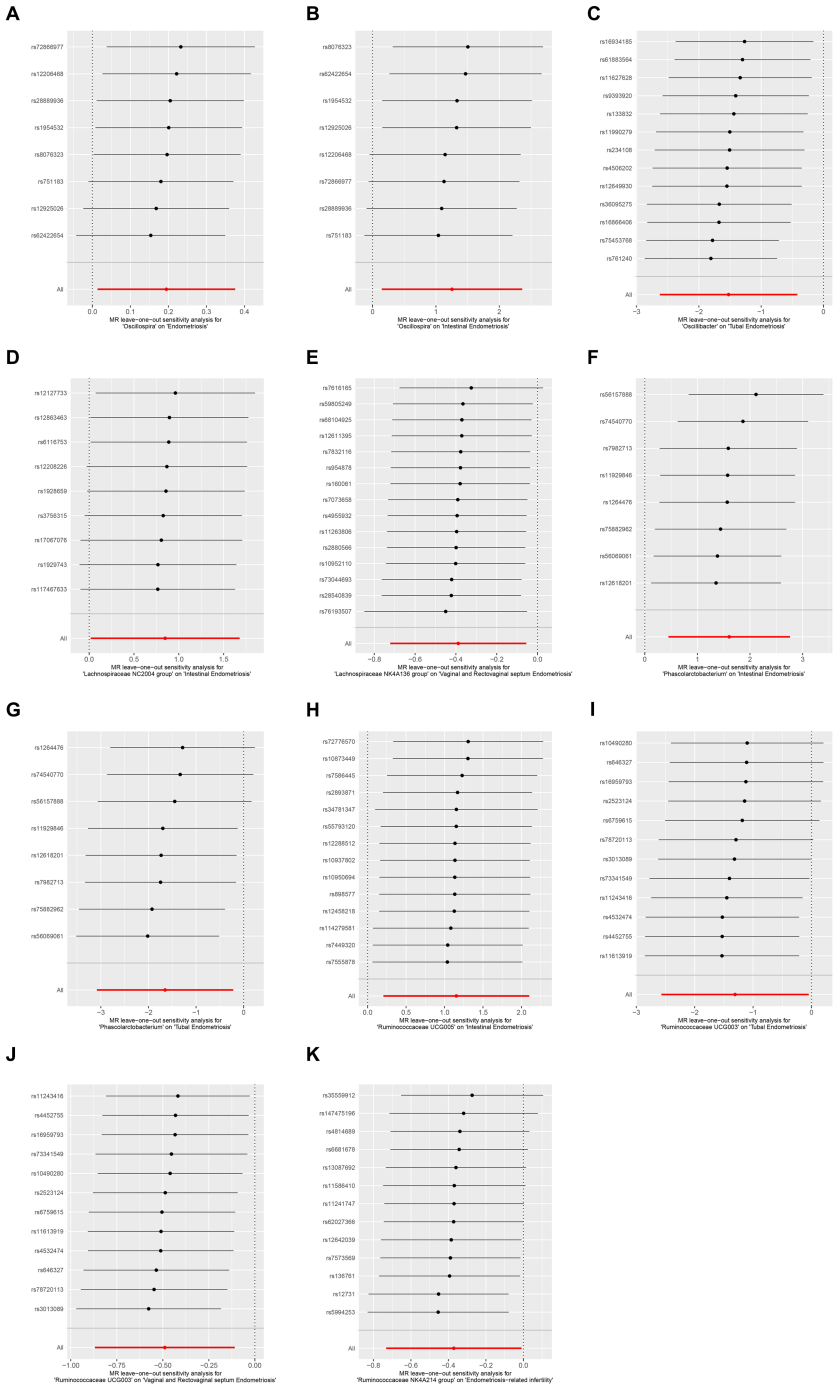
Leave-one-out sensitivity analysis demonstrated the opposite effect of the gut microbiome at the family level in different subtypes of endometriosis. **(A)** MR leave-one-out sensitivity analysis for *Oscillospira* on endometriosis. **(B)** MR leave-one-out sensitivity analysis for *Oscillospira* on intestinal endometriosis. **(C)** MR leave-one-out sensitivity analysis for *Oscillospira* on tubal endometriosis. **(D)** MR leave-one-out sensitivity analysis for *Lachnospiraceae NC2004* on intestinal endometriosis. **(E)** MR leave-one-out sensitivity analysis for *Lachnospiraceae NK4A136* group on vaginal and rectovaginal septum endometriosis. **(F)** MR leave-one-out sensitivity analysis for *Phascolarctobacterium* on intestinal endometriosis. **(G)** MR leave-one-out sensitivity analysis for *Phascolarctobacterium* on tubal endometriosis. **(H)** MR leave-one-out sensitivity analysis for *Ruminococcaceae UCG005* on intestinal endometriosis. **(I)** MR leave-one-out sensitivity analysis for *Ruminococcaceae UCG003* on tubal endometriosis. **(J)** MR leave-one-out sensitivity analysis for *Ruminococcaceae UCG003* on vaginal and rectovaginal septum endometriosis. **(K)** MR leave-one-out sensitivity analysis for *Ruminococcaceae NK4A214* group on endometriosis-related infertility.

We further sought to investigate the associations between gut microbiota and co-occurrence of endometriosis and infertility. Three significant associations at the genus level were identified. The *Actinomyces* genus was positively associated with the risk of endometriosis-related infertility (OR: 1.657, 95% CI: 1.187–2.312, *p* = 0.00298). Conversely, the *Holdemania* genus (OR: 0.63, 95% CI: 0.444–0.894, *p* = 0.00969) and *Ruminococcaceae NK4A214* group (OR: 0.69, 95% CI: 0.481–0.99, *p* = 0.0439) were negatively associated with the risk of the simultaneous diagnosis of endometriosis and infertility (Figure 2 and Table 2). The leave-one-out results further confirmed the robustness of our data (Figures 3, 4).

## 4 Discussion

Despite the growing research interest in the relationships between gut microbiota and endometriosis, the underlying causes and mechanisms of endometriosis remain unclear. Our study represents the first attempt to investigate the associations between gut microbiota and endometriosis at various sites, including the uterus, ovary, fallopian tubes, pelvic peritoneum, vagina, and rectovaginal septum, as well as the intestine, using the two-sample MR analysis. Moreover, our study is the first to explore the association between gut microbiota at the genus level and endometriosis in women with infertility. Several associations between gut microbiota and various subtypes of endometriosis at different anatomical sites were recognized. Specifically, three, six, four, five, and seven genera were associated with ovarian, tubal, pelvic peritoneum, vaginal/rectovaginal septum, and intestinal endometriosis, respectively. Additionally, one genus was associated with adenomyosis, and three genera were linked with endometriosis-related infertility. This research offers novel insights into endometriosis mechanisms and potential directions for treating endometriosis in women with infertility.

Previous MR research has highlighted a relationship between the gut microbiome and endometriosis. Liang et al. (2023) identified *Anaerotruncus*, *Olsenella*, and *Oscillospira* genera as harmful factors in endometriosis this result is in accordance with our findings. The researchers proposed that *Olsenella* may be implicated in the pathogenesis of endometriosis through the regulation of Interleukin 10 (IL-10) levels. In addition, Liu et al. (2023) identified *Anaerotruncus* as a risk factor for ovarian endometriosis. Walther-Antonio et al. (2016) reported remarkable concentrations of *Anaerotruncus* in patients with endometrial cancer. Furthermore, *Anaerotruncus* was found to be positively correlated with glucose intolerance and increased intestinal permeability (Kong et al., 2019). However, the mechanism of its involvement in the development of endometriosis remains unknown.

Notably, our study indicates that the *Eubacterium ruminantium* group may act as a protective factor against endometriosis. Moreover, the *Eubacterium oxidoreducens* group has been identified as a protective factor against vaginal and rectovaginal septum endometriosis and pelvic peritoneal endometriosis at the genus level. The *Eubacterium ruminantium* group and *Eubacterium oxidoreducens* produce butyric acid, a short-chain fatty acid (SCFA) (Krumholz et al., 1987; Maia et al., 2007). SCFAs, encompassing butyrate, acetic acid, and propionate, are metabolic byproducts of undigested dietary fiber fermented by gut microbiota. They not only supply energy to the host and intestinal epithelial cells, but they also counteract the invasion of intestinal pathogens and safeguard the function of the intestinal mucosal barrier. A decrease in SCFA concentration in the human gut can precipitate disease onset. Previous research has demonstrated that in mice with endometriosis, fecal butyrate levels are diminished, and butyrate application curtails the growth of endometriotic lesions (Chadchan et al., 2021). SCFAs primarily exert anti-inflammatory effects through two signaling pathways (Guo and Zhang, 2024). First, they activate G protein-coupled receptors such as GPR41, GPR43, and GPR109a, inhibiting the activation of Nod-like receptor pyrin domain 3 inflammasomes and thus reducing the secretion of proinflammatory cytokines. Second, they can also inhibit histone deacetylases, reducing the production of the proinflammatory tumor necrosis factor, and leading to the inactivation of nuclear factor kappa B. Additionally, research has found that butyrate can modify the cytokines of helper T cells and foster the integrity of the intestinal epithelial barrier (Kau et al., 2011). Our results further suggest that the *Eubacterium* genus may stave off endometriosis by participating in the regulation of immune responses, providing fresh insights for the treatment of endometriosis.

Our research findings suggest that the *Holdemania* genus not only plays a protective role in adenomyosis but also in endometriosis-associated infertility. Our conclusion aligns with that of a previous study that identified *Holdemania* as a protective factor against female infertility (Xi et al., 2023), thus reinforcing the validity of our results. The frequent co-occurrence of infertility and endometriosis suggests a close correlation between these conditions. The mechanisms of inflammation and immune dysregulation are now widely accepted to negatively affect endometrial receptivity and embryonic viability at implantation sites. Current literature indicates that chronic inflammation associated with endometriosis can instigate alterations in the endometrial microenvironment and immune milieu (Vallve-Juanico et al., 2019). Immune-mediated events are crucial factors in endometriosis-associated infertility (Kolanska et al., 2021). The presence of either endometriosis or autoimmune diseases, or a combination thereof, can amplify inflammation and immune activity, potentially impairing oocyte maturation, embryo development, and endometrial receptivity; this effect disrupts endometrium-embryo crosstalk at the implantation site (Salmeri et al., 2023a). Gut microbiota can influence female fertility by fostering an inflammatory environment in the reproductive tract, abdominal cavity, and pelvis (Salliss et al., 2021). Prior research has identified the increased expression of *Holdemania* in other inflammatory diseases (Jang et al., 2020). Moreover, rodent studies have shown that the consumption of sucrose and fructose can alter microbiota composition, leading to the proliferation of *Holdemania* and the onset of colitis (Song et al., 2023). In a mouse model of carbapenem-resistant *Klebsiella pneumonia*, the administration of *Lactobacillus LP1812* resulted in an increased abundance of *Holdemania*, underscoring its potential role as a gut probiotic (Yan et al., 2021). The *Lactobacillus* genus has been revealed to mitigate endometriotic lesions in mice by enhancing the levels of IL-12 and the activity of natural killer cells (Itoh et al., 2011).

Women with endometriosis are two to three times more likely to be diagnosed with IBS, with over 20% of patients with endometriosis presenting IBS-like symptoms (Chiaffarino et al., 2021). Extant research has found that gut microbiota contributes to the pathogenesis of IBS and endometriosis via similar mechanisms. Gut microbiota dysbiosis can affect intestinal permeability, initiate inflammatory responses, and provoke immune reactions. Furthermore, both conditions are linked to the central and enteric nervous systems via a bidirectional communication pathway known as the gut-brain axis, which regulates secretion, immune responses, intestinal motility, and visceral sensitivity (Salmeri et al., 2023b). Therefore, we propose that the *Holdemania* genus may be involved in the pathogenesis of adenomyosis and endometriosis-associated infertility through inflammatory and immune responses. Additionally, we identified *Barnesiella* as a protective factor against endometriosis affecting the ovaries and fallopian tubes. Previous studies have reported an association between *Barnesiella* and variation in the bilirubin reductase A locus (Ruhlemann et al., 2021). Biliverdin reductase A has been proven to suppress the expression of the TLR4 gene, a pattern recognition receptor that modulates intracellular immune and inflammatory responses and is ubiquitous on immune cell membranes. LPS can activate Nuclear Factor Kappa-B (NF-kB) via TLR4 binding and MyD88-dependent and -independent pathways (Khan et al., 2018), thereby eliciting immune and inflammatory responses and promoting the transcription of genes associated with cell adhesion, proliferation, and antiapoptosis (Guo and Zhang, 2024). Therefore, we postulate that *Barnesiella* may exert a protective effect against endometriosis by modulating the LPS/TLR4/NF-kB pathway to inhibit the initiation and progression of endometriosis.

Additionally, our study found that the same genus can play contrasting roles at different sites. Specifically, the *Phascolarctobacterium* genus, while identified as a protective factor against tubal endometriosis, posed a risk for intestinal endometriosis. Previous research has shown that *Phascolarctobacterium* can metabolize succinic acid to produce propionate and acetic acid. Succinic acid can activate immune cells via the specific surface receptor succinic acid receptor 1, thereby enhancing inflammatory responses (Connors et al., 2018). Propionic acid, on the other hand, is recognized as an effective immunomodulatory supplement that augments the function of intestinal T-regulatory cells associated with multiple sclerosis (Duscha et al., 2020). Propionate has also demonstrated anti-inflammatory effects in human subcutaneous adipose tissue (Al-Lahham and Rezaee, 2019). This dichotomy may originate from the complex balance between pro- and anti-inflammatory metabolic signals involving succinate as an intermediate and SCFAs. Therefore, the *Phascolarctobacterium* genus has diverse roles at various sites of endometriosis. Moreover, two members of the same family, namely, *Lachnospiraceae NC2004* and *Lachnospiraceae NK4A136*, play contrasting roles. The former is a risk factor for intestinal endometriosis, whereas the latter is a protective factor against vaginal and rectovaginal septum endometriosis. *Lachnospiraceae* members are major producers of SCFAs and are implicated in various inflammatory diseases (Vacca et al., 2020). *Ruminococcaceae UCG003*, *Ruminococcaceae NK4A214* group, and *Ruminococcaceae UCG005* are all from the same family. The first two exert protective effects against the fallopian tube, vagina, and rectovaginal septum endometriosis, as well as endometriosis-related infertility, whereas the third poses a risk for intestinal endometriosis. Past research has established a causal link between *Ruminococcaceae* and endometriosis (Ji et al., 2023). *Ruminococcaceae* is known primarily for producing acetic acid (Chassard and Bernalier-Donadille, 2006), and some members follow a succinate pathway with succinate as the end product instead of propionate (Louis and Flint, 2017). Ji et al. (2023) suggested that the divergent abilities of *Ruminococcaceae* members to produce butyrate might account for the contrasting causal relationships within the *Ruminococcaceae* family. Further investigation is required to explore these aspects in the future.

The association between gut microbiota and endometriosis has been the focus of extensive research. In our study, we used MR analysis to investigate the associations between gut microbiota and endometriosis in different regions based on GWAS summary statistics. Rigorous quality controls were used to eliminate IVs that could potentially bias the results. The MR approach offers an advantage over traditional observational studies because it is less susceptible to confounding factors than other approaches. However, our study has some limitations. The precise biological functions of numerous genetic variants remain unknown, and MR may be prone to errors if genetic variants are pleiotropic. Moreover, population stratification could bias the results. Our gut microbiota data included participants of multiple races and both sexes, whereas endometriosis datasets were collected from European women. However, due to the absence of demographic data in the original study, we could not circumvent this bias by performing gender or ethnic subgroup analyses. The GWAS dataset contains cases wherein multiple locations are concurrently afflicted by endometriosis. Owing to the absence of raw data for these cases, we were unable to conduct further analyses and exclusions. Furthermore, the smaller sample size per taxon in gut microbiota (GWAS) than that in complex diseases leads to the insufficient identification of IVs at the genus level. Finally, we did not conduct bidirectional MR analysis due to the limited number of SNP sites for outcomes, and bidirectional causality between exposure and outcome cannot be excluded.

In conclusion, our study elucidates relationships between gut microbiota and site-specific endometriosis, thereby augmenting our understanding of the pathophysiology of endometriosis. Moreover, our findings pave the way for potential therapeutic strategies targeting gut microbiota for individuals grappling with endometriosis-related infertility. In the future, further validation analyses will be performed on animal models of endometriosis and human gut microbiota samples.

## Data availability statement

The datasets presented in this study can be found in online repositories. The names of the repository/repositories and accession number(s) can be found in the article/Supplementary material.

## Ethics statement

The studies involving humans were approved by the Institutional Review Board. Data used in this study are publicly accessible, de-identified. Hence, no additional ethical approval was required. The studies were conducted in accordance with the local legislation and institutional requirements. Written informed consent for participation in this study was provided by the participants’ legal guardians/next of kin.

## Author contributions

YT: Visualization, Data curation, Writing – review & editing, Writing – original draft. JY: Visualization, Data curation, Writing – review & editing, Writing – original draft. FH: Writing – review & editing, Writing – original draft, Visualization, Data curation. HH: Writing – review & editing, Writing – original draft, Visualization, Data curation. LJ: Writing – review & editing, Writing – original draft, Supervision, Data curation.

## Footnotes

^1^https://gwas.mrcieu.ac.uk/

## References

[ref1] Al-Lahham S. Rezaee F. (2019). Propionic acid counteracts the inflammation of human subcutaneous adipose tissue: a new avenue for drug development. Daru 27, 645–652. doi: 10.1007/s40199-019-00294-z, PMID: 31512194 PMC6895314

[ref2] Anderson G. (2019). Endometriosis pathoetiology and pathophysiology: roles of vitamin A, estrogen, immunity, adipocytes, gut microbiome and melatonergic pathway on mitochondria regulation. Biomol. Concepts 10, 133–149. doi: 10.1515/bmc-2019-0017, PMID: 31339848

[ref3] Baker J. M. Al-Nakkash L. Herbst-Kralovetz M. M. (2017). Estrogen-gut microbiome axis: physiological and clinical implications. Maturitas 103, 45–53. doi: 10.1016/j.maturitas.2017.06.025, PMID: 28778332

[ref4] Becker C. M. Bokor A. Heikinheimo O. Horne A. Jansen F. Kiesel L.. (2022). ESHRE guideline: endometriosis. Hum. Reprod. Open 2022:hoac009. doi: 10.1093/hropen/hoac009, PMID: 35350465 PMC8951218

[ref5] Bowden J. Davey S. G. Burgess S. (2015). Mendelian randomization with invalid instruments: effect estimation and bias detection through Egger regression. Int. J. Epidemiol. 44, 512–525. doi: 10.1093/ije/dyv080, PMID: 26050253 PMC4469799

[ref6] Bowden J. Davey S. G. Haycock P. C. Burgess S. (2016). Consistent estimation in Mendelian randomization with some invalid instruments using a weighted median estimator. Genet. Epidemiol. 40, 304–314. doi: 10.1002/gepi.21965, PMID: 27061298 PMC4849733

[ref7] Bulun S. E. Yilmaz B. D. Sison C. Miyazaki K. Bernardi L. Liu S.. (2019). Endometriosis. Endocr. Rev. 40, 1048–1079. doi: 10.1210/er.2018-00242, PMID: 30994890 PMC6693056

[ref8] Burgess S. Butterworth A. Thompson S. G. (2013). Mendelian randomization analysis with multiple genetic variants using summarized data. Genet. Epidemiol. 37, 658–665. doi: 10.1002/gepi.21758, PMID: 24114802 PMC4377079

[ref9] Burgess S. Small D. S. Thompson S. G. (2017). A review of instrumental variable estimators for Mendelian randomization. Stat. Methods Med. Res. 26, 2333–2355. doi: 10.1177/0962280215597579, PMID: 26282889 PMC5642006

[ref10] Burgess S. Thompson S. G. (2011). Avoiding bias from weak instruments in Mendelian randomization studies. Int. J. Epidemiol. 40, 755–764. doi: 10.1093/ije/dyr036, PMID: 21414999

[ref11] Burgess S. Thompson S. G. (2017). Interpreting findings from Mendelian randomization using the MR-Egger method. Eur. J. Epidemiol. 32, 377–389. doi: 10.1007/s10654-017-0255-x, PMID: 28527048 PMC5506233

[ref12] Chadchan S. B. Popli P. Ambati C. R. Tycksen E. Han S. J. Bulun S. E.. (2021). Gut microbiota-derived short-chain fatty acids protect against the progression of endometriosis. Life Sci. Alliance 4:e202101224. doi: 10.26508/lsa.202101224, PMID: 34593556 PMC8500332

[ref13] Chassard C. Bernalier-Donadille A. (2006). H2 and acetate transfers during xylan fermentation between a butyrate-producing xylanolytic species and hydrogenotrophic microorganisms from the human gut. FEMS Microbiol. Lett. 254, 116–122. doi: 10.1111/j.1574-6968.2005.00016.x, PMID: 16451188

[ref14] Chiaffarino F. Cipriani S. Ricci E. Mauri P. A. Esposito G. Barretta M.. (2021). Endometriosis and irritable bowel syndrome: a systematic review and meta-analysis. Arch. Gynecol. Obstet. 303, 17–25. doi: 10.1007/s00404-020-05797-8, PMID: 32949284

[ref15] Connors J. Dawe N. Van Limbergen J. (2018). The role of succinate in the regulation of intestinal inflammation. Nutrients 11:25. doi: 10.3390/nu11010025, PMID: 30583500 PMC6356305

[ref16] Duscha A. Gisevius B. Hirschberg S. Yissachar N. Stangl G. I. Dawin E.. (2020). Propionic acid shapes the multiple sclerosis disease course by an immunomodulatory mechanism. Cell 180, 1067–1080.e16. doi: 10.1016/j.cell.2020.02.035, PMID: 32160527

[ref17] Ervin S. M. Li H. Lim L. Roberts L. R. Liang X. Mani S.. (2019). Gut microbial beta-glucuronidases reactivate estrogens as components of the estrobolome that reactivate estrogens. J. Biol. Chem. 294, 18586–18599. doi: 10.1074/jbc.RA119.010950, PMID: 31636122 PMC6901331

[ref18] Greco M. F. Minelli C. Sheehan N. A. Thompson J. R. (2015). Detecting pleiotropy in Mendelian randomisation studies with summary data and a continuous outcome. Stat. Med. 34, 2926–2940. doi: 10.1002/sim.6522, PMID: 25950993

[ref19] Grover S. Del G. M. F. Stein C. M. Ziegler A. (2017). Mendelian randomization. Methods Mol. Biol. 1666, 581–628. doi: 10.1007/978-1-4939-7274-6_29 28980266

[ref20] Guo C. Zhang C. (2024). Role of the gut microbiota in the pathogenesis of endometriosis: a review. Front. Microbiol. 15:1363455. doi: 10.3389/fmicb.2024.1363455, PMID: 38505548 PMC10948423

[ref21] Hartwig F. P. Davey S. G. Bowden J. (2017). Robust inference in summary data Mendelian randomization via the zero modal pleiotropy assumption. Int. J. Epidemiol. 46, 1985–1998. doi: 10.1093/ije/dyx102, PMID: 29040600 PMC5837715

[ref22] Itoh H. Sashihara T. Hosono A. Kaminogawa S. Uchida M. (2011). *Lactobacillus gasseri* oll2809 inhibits development of ectopic endometrial cell in peritoneal cavity via activation of NK cells in a murine endometriosis model. Cytotechnology 63, 205–210. doi: 10.1007/s10616-011-9343-z, PMID: 21409454 PMC3080482

[ref23] Jang J. H. Yeom M. J. Ahn S. Oh J. Y. Ji S. Kim T. H.. (2020). Acupuncture inhibits neuroinflammation and gut microbial dysbiosis in a mouse model of Parkinson’s disease. Brain Behav. Immun. 89, 641–655. doi: 10.1016/j.bbi.2020.08.015, PMID: 32827699

[ref24] Javert C. T. (1949). Pathogenesis of endometriosis based on endometrial homeoplasia, direct extension, exfoliation and implantation, lymphatic and hematogenous metastasis, including five case reports of endometrial tissue in pelvic lymph nodes. Cancer 2, 399–410. doi: 10.1002/1097-0142(194905)2:3<399::aid-cncr2820020304>3.0.co;2-l, PMID: 18131400

[ref25] Ji X. Yang Q. Zhu X. L. Xu L. Guo J. Y. Rong Y.. (2023). Association between gut microbiota and endometriosis: a two-sample Mendelian randomization study. Front. Microbiol. 14:1188458. doi: 10.3389/fmicb.2023.1188458, PMID: 37829443 PMC10565803

[ref26] Kau A. L. Ahern P. P. Griffin N. W. Goodman A. L. Gordon J. I. (2011). Human nutrition, the gut microbiome and the immune system. Nature 474, 327–336. doi: 10.1038/nature10213, PMID: 21677749 PMC3298082

[ref27] Khan K. N. Fujishita A. Hiraki K. Kitajima M. Nakashima M. Fushiki S.. (2018). Bacterial contamination hypothesis: a new concept in endometriosis. Reprod. Med. Biol. 17, 125–133. doi: 10.1002/rmb2.12083, PMID: 29692669 PMC5902457

[ref28] Kolanska K. Alijotas-Reig J. Cohen J. Cheloufi M. Selleret L. D’Argent E.. (2021). Endometriosis with infertility: a comprehensive review on the role of immune deregulation and immunomodulation therapy. Am. J. Reprod. Immunol. 85:e13384. doi: 10.1111/aji.13384, PMID: 33278837

[ref29] Kong C. Gao R. Yan X. Huang L. Qin H. (2019). Probiotics improve gut microbiota dysbiosis in obese mice fed a high-fat or high-sucrose diet. Nutrition 60, 175–184. doi: 10.1016/j.nut.2018.10.002 30611080

[ref30] Krumholz L. R. Crawford R. L. Hemling M. E. Bryant M. P. (1987). Metabolism of gallate and phloroglucinol in *Eubacterium oxidoreducens* via 3-hydroxy-5-oxohexanoate. J. Bacteriol. 169, 1886–1890. doi: 10.1128/jb.169.5.1886-1890.1987, PMID: 3571153 PMC212039

[ref31] Kurilshikov A. Medina-Gomez C. Bacigalupe R. Radjabzadeh D. Wang J. Demirkan A.. (2021). Large-scale association analyses identify host factors influencing human gut microbiome composition. Nat. Genet. 53, 156–165. doi: 10.1038/s41588-020-00763-1, PMID: 33462485 PMC8515199

[ref32] Kwa M. Plottel C. S. Blaser M. J. Adams S. (2016). The intestinal microbiome and estrogen receptor-positive female breast cancer. J. Natl. Cancer Inst. 108:djw029. doi: 10.1093/jnci/djw029, PMID: 27107051 PMC5017946

[ref33] Lagana A. S. Vitale S. G. Salmeri F. M. Triolo O. Ban F. H. Vrtacnik-Bokal E.. (2017). Unus pro omnibus, omnes pro uno: a novel, evidence-based, unifying theory for the pathogenesis of endometriosis. Med. Hypotheses 103, 10–20. doi: 10.1016/j.mehy.2017.03.032, PMID: 28571791

[ref34] Liang Z. Di N. Li L. Yang D. (2021). Gut microbiota alterations reveal potential gut-brain axis changes in polycystic ovary syndrome. J. Endocrinol. Investig. 44, 1727–1737. doi: 10.1007/s40618-020-01481-5, PMID: 33387350

[ref35] Liang Y. Zeng W. Hou T. Yang H. Wu B. Pan R.. (2023). Gut microbiome and reproductive endocrine diseases: a Mendelian randomization study. Front. Endocrinol. 14:1164186. doi: 10.3389/fendo.2023.1164186, PMID: 37600687 PMC10436605

[ref36] Liu Z. Chen P. Luo L. Liu Q. Shi H. Yang X. (2023). Causal effects of gut microbiome on endometriosis: a two-sample Mendelian randomization study. BMC Womens Health 23:637. doi: 10.1186/s12905-023-02742-0, PMID: 38037013 PMC10687921

[ref37] Louis P. Flint H. J. (2017). Formation of propionate and butyrate by the human colonic microbiota. Environ. Microbiol. 19, 29–41. doi: 10.1111/1462-2920.13589 27928878

[ref38] Machairiotis N. Stylianaki A. Dryllis G. Zarogoulidis P. Kouroutou P. Tsiamis N.. (2013). Extrapelvic endometriosis: a rare entity or an under diagnosed condition? Diagn. Pathol. 8:194. doi: 10.1186/1746-1596-8-194, PMID: 24294950 PMC3942279

[ref39] Maia M. R. Chaudhary L. C. Figueres L. Wallace R. J. (2007). Metabolism of polyunsaturated fatty acids and their toxicity to the microflora of the rumen. Antonie Van Leeuwenhoek 91, 303–314. doi: 10.1007/s10482-006-9118-2, PMID: 17072533

[ref40] Novello M. Mandarino F. V. Di Saverio S. Gori D. Lugaresi M. Duchi A.. (2019). Post-operative outcomes and predictors of mortality after colorectal cancer surgery in the very elderly patients. Heliyon 5:e02363. doi: 10.1016/j.heliyon.2019.e02363, PMID: 31485540 PMC6716468

[ref41] Ozkan S. Murk W. Arici A. (2008). Endometriosis and infertility: epidemiology and evidence-based treatments. Ann. N. Y. Acad. Sci. 1127, 92–100. doi: 10.1196/annals.1434.007, PMID: 18443335

[ref42] Qi X. Yun C. Pang Y. Qiao J. (2021). The impact of the gut microbiota on the reproductive and metabolic endocrine system. Gut Microbes 13, 1–21. doi: 10.1080/19490976.2021.1894070, PMID: 33722164 PMC7971312

[ref43] Rinninella E. Raoul P. Cintoni M. Franceschi F. Miggiano G. Gasbarrini A.. (2019). What is the healthy gut microbiota composition? A changing ecosystem across age, environment, diet, and diseases. Microorganisms 7:14. doi: 10.3390/microorganisms7010014, PMID: 30634578 PMC6351938

[ref44] Ruhlemann M. C. Hermes B. M. Bang C. Doms S. Moitinho-Silva L. Thingholm L. B.. (2021). Genome-wide association study in 8,956 German individuals identifies influence of ABO histo-blood groups on gut microbiome. Nat. Genet. 53, 147–155. doi: 10.1038/s41588-020-00747-1, PMID: 33462482

[ref45] Salliss M. E. Farland L. V. Mahnert N. D. Herbst-Kralovetz M. M. (2021). The role of gut and genital microbiota and the estrobolome in endometriosis, infertility and chronic pelvic pain. Hum. Reprod. Update 28, 92–131. doi: 10.1093/humupd/dmab035, PMID: 34718567

[ref46] Salmeri N. Gennarelli G. Vanni V. S. Ferrari S. Ruffa A. Rovere-Querini P.. (2023a). Concomitant autoimmunity in endometriosis impairs endometrium-embryo crosstalk at the implantation site: a multicenter case-control study. J. Clin. Med. 12:3557. doi: 10.3390/jcm12103557, PMID: 37240662 PMC10219087

[ref47] Salmeri N. Sinagra E. Dolci C. Buzzaccarini G. Sozzi G. Sutera M.. (2023b). Microbiota in irritable bowel syndrome and endometriosis: birds of a feather flock together-a review. Microorganisms 11:2089. doi: 10.3390/microorganisms11082089, PMID: 37630649 PMC10458414

[ref48] Sampson J. (1927). Peritoneal endometriosis due to the menstrual dissemination of endometrial tissue into the peritoneal cavity. Am. J. Obstet. Gynecol. 14, 422–469. doi: 10.1016/S0002-9378(15)30003-X

[ref49] Sampson J. A. (1940). The development of the implantation theory for the origin of peritoneal endometriosis. Am. J. Obstet. Gynecol. 40, 549–557. doi: 10.1016/S0002-9378(40)91238-8

[ref50] Saunders P. Horne A. W. (2021). Endometriosis: etiology, pathobiology, and therapeutic prospects. Cell 184, 2807–2824. doi: 10.1016/j.cell.2021.04.041, PMID: 34048704

[ref51] Song G. Gan Q. Qi W. Wang Y. Xu M. Li Y. (2023). Fructose stimulated colonic arginine and proline metabolism dysbiosis, altered microbiota and aggravated intestinal barrier dysfunction in DSS-induced colitis rats. Nutrients 15:782. doi: 10.3390/nu15030782, PMID: 36771488 PMC9921751

[ref52] Talwar C. Singh V. Kommagani R. (2022). The gut microbiota: a double-edged sword in endometriosis. Biol. Reprod. 107, 881–901. doi: 10.1093/biolre/ioac147, PMID: 35878972 PMC9562115

[ref53] Tang F. Deng M. Xu C. Yang R. Ji X. Hao M.. (2024). Unraveling the microbial puzzle: exploring the intricate role of gut microbiota in endometriosis pathogenesis. Front. Cell. Infect. Microbiol. 14:1328419. doi: 10.3389/fcimb.2024.1328419, PMID: 38435309 PMC10904627

[ref54] Taylor H. S. Kotlyar A. M. Flores V. A. (2021). Endometriosis is a chronic systemic disease: clinical challenges and novel innovations. Lancet 397, 839–852. doi: 10.1016/S0140-6736(21)00389-5, PMID: 33640070

[ref55] Vacca M. Celano G. Calabrese F. M. Portincasa P. Gobbetti M. De Angelis M. (2020). The controversial role of human gut *Lachnospiraceae*. Microorganisms 8:573. doi: 10.3390/microorganisms8040573, PMID: 32326636 PMC7232163

[ref56] Vallve-Juanico J. Houshdaran S. Giudice L. C. (2019). The endometrial immune environment of women with endometriosis. Hum. Reprod. Update 25, 565–592. doi: 10.1093/humupd/dmz018, PMID: 31424502 PMC6737540

[ref57] Vercellini P. Vigano P. Somigliana E. Fedele L. (2014). Endometriosis: pathogenesis and treatment. Nat. Rev. Endocrinol. 10, 261–275. doi: 10.1038/nrendo.2013.255 24366116

[ref58] Walther-Antonio M. R. Chen J. Multinu F. Hokenstad A. Distad T. J. Cheek E. H.. (2016). Potential contribution of the uterine microbiome in the development of endometrial cancer. Genome Med. 8:122. doi: 10.1186/s13073-016-0368-y, PMID: 27884207 PMC5123330

[ref59] Wang Y. Nicholes K. Shih I. M. (2020). The origin and pathogenesis of endometriosis. Annu. Rev. Pathol. 15, 71–95. doi: 10.1146/annurev-pathmechdis-012419-032654, PMID: 31479615 PMC7980953

[ref60] Xholli A. Cremonini F. Perugi I. Londero A. P. Cagnacci A. (2023). Gut microbiota and endometriosis: exploring the relationship and therapeutic implications. Pharmaceuticals 16:1696. doi: 10.3390/ph16121696, PMID: 38139822 PMC10747908

[ref61] Xi Y. Zhang C. Feng Y. Zhao S. Zhang Y. Duan G.. (2023). Genetically predicted the causal relationship between gut microbiota and infertility: bidirectional Mendelian randomization analysis in the framework of predictive, preventive, and personalized medicine. EPMA J. 14, 405–416. doi: 10.1007/s13167-023-00332-6, PMID: 37605651 PMC10439866

[ref62] Yan R. Lu Y. Wu X. Yu P. Lan P. Wu X.. (2021). Anticolonization of carbapenem-resistant *Klebsiella pneumoniae* by *Lactobacillus plantarum* lp1812 through accumulated acetic acid in mice intestinal. Front. Cell. Infect. Microbiol. 11:804253. doi: 10.3389/fcimb.2021.804253, PMID: 34976873 PMC8714838

[ref63] Yang G. Wei J. Liu P. Zhang Q. Tian Y. Hou G.. (2021). Role of the gut microbiota in type 2 diabetes and related diseases. Metabolism 117:154712. doi: 10.1016/j.metabol.2021.154712 33497712

